# Assessment of Patient Knowledge, Awareness, and Adherence in Heart Failure in a Real-Life Setting: Insights from Data Acquired in Pharmacies

**DOI:** 10.3390/jcm11030863

**Published:** 2022-02-07

**Authors:** Masatake Kobayashi, Christophe Wilcke, Nicolas Girerd

**Affiliations:** 1 Université de Lorraine, Inserm, Centre d’Investigations Cliniques 1433 and Inserm U1116, CHRU Nancy, F-CRIN INI-CRCT, 54500 Nancy, France; mkoba12@me.com; 2 Department of Cardiology, Tokyo Medical University, Tokyo 160-8402, Japan; 3 Union Régionale des Professionnels de Santé Pharmaciens, 54000 Nancy, France; agnes.wilcke-clement@wanadoo.fr; 4 Département de Cardiologie, CHRU Nancy, 54500 Nancy, France

**Keywords:** heart failure, patient adherence, education, cardiovascular diseases, cardiac edema

## Abstract

Background: Patient knowledge is crucial for managing and/or monitoring patients with heart failure (HF). However, “real-life” evidence of knowledge level and awareness in HF is yet to be explored. We assessed unselected HF patients’ knowledge and awareness in a pharmacy setting. Methods: One hundred eight HF patients (mean age [SD], 70 (12) years, 61% men) were studied in pharmacies in the north-east region of France in 2019. All patients were interviewed by their pharmacist to quantify their knowledge in HF, self-assessment of symptoms of congestion, as well as their adherence to HF treatment and guideline-recommended lifestyle. Results: Overall, 40% of patients had not consulted their cardiologist in the past 6 months, and 89% never underwent an HF education program. Regarding HF knowledge, nearly half were unsure whether they had HF (43.5%). Only half of the patients knew how to self-assess HF symptoms (57.4%), while a quarter (25%) were unsure of the purpose of HF medications. Conclusions: In patients with HF assessed in their pharmacies, a majority lacked fundamental knowledge regarding HF, such as self-assessment of congestion, possibly due to a minimal proportion of patients undergoing an HF education program. These results suggest that interventions led by pharmacies may help improve HF education coverage in patients who may have poor access to specialized care.

## 1. Introduction

Recurrent hospitalizations for worsening heart failure (HF) are common [1] and possibly result from disease progression and/or suboptimal treatment. However, poor diet and/or a lack of patient education and awareness may also increase the risk of worsening HF [2,3]. In addition, poor access to health care services due to geographical barriers may constitute hurdles for appropriate HF management [4]. Epidemiological data in this field may also suffer from inclusion bias since investigators working in centers more prone to participate in these studies are likely to better educate and manage their patients. Data arising from “real life” settings in order to better assess the level of HF awareness and patient education are warranted.

Pharmacies are unique settings to acquire data from unselected patients with HF since these patients require a regular pharmacy visit for their disease-modifying treatments. Data assessed by pharmacies might consequently provide unbiased estimates of HF patients’ knowledge/awareness.

We aimed to assess patients’ knowledge of disease progression, self-assessment, lifestyle, and/or purpose of medications in pharmacies.

## 2. Methods

The present study enrolled 108 patients with HF aged 60 years or older in pharmacies in the northeast region of France between August 2019 and September 2020. HF diagnosis was defined by the guidelines of the European Society of Cardiology [5]. Based on our algorithm, we included patients that had either a history of HF hospitalization, which was registered by the national social security system, or were treated with angiotensin receptor neprilysin inhibitor, which was the specific treatment for HF. This study used completely anonymized data acquired within routine practice and consequently did not require ethics committee approval.

Pharmacists interviewed all patients with a short questionnaire, focusing on HF within an HF awareness initiative. The questionnaire assessed their knowledge regarding disease progression, adherence to treatment, education, and how to self-manage signs and symptoms of congestion (Supplementary Figure S1). The answers provided in this questionnaire help pharmacists in providing educational information related to HF tailored to the patient’s profile. The data collected from these questionnaires were then anonymized and used in the analysis reported herein.

Continuous variables are described as means ± standard deviation and categorical variables as frequencies (percentages).

## 3. Results

**Figure 1:**
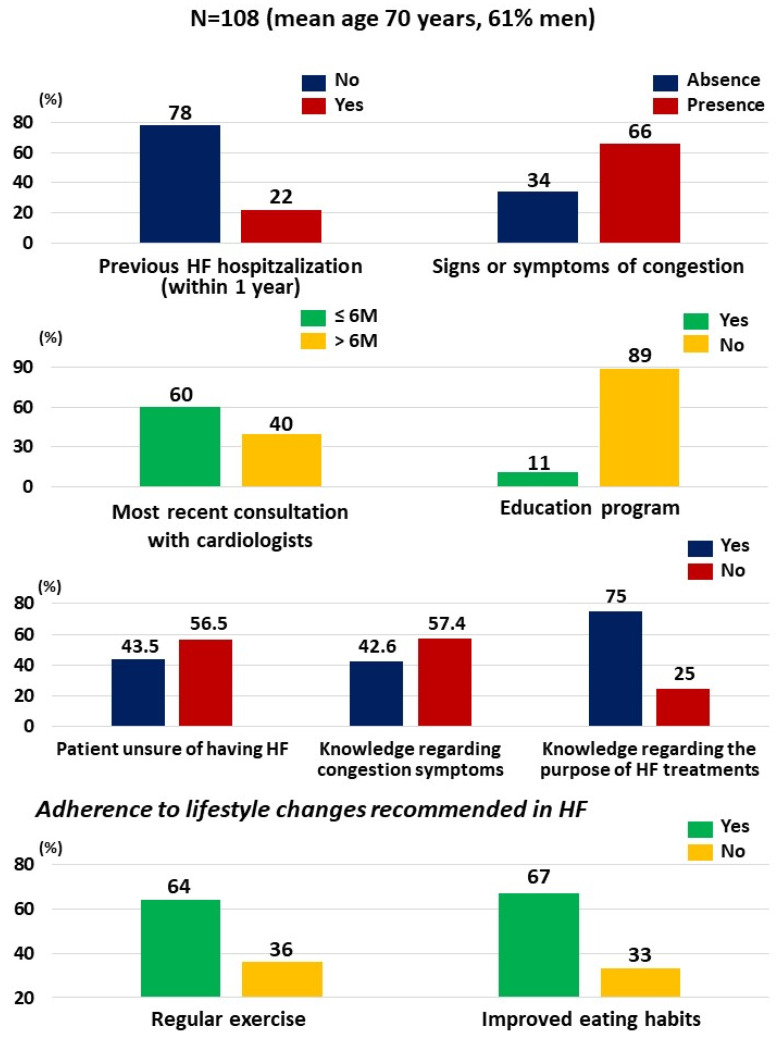
Knowledge, awareness, and adherence of unselected HF patients interviewed in pharmacies. HF, heart failure; M, months.

The results regarding knowledge, awareness, and adherence of included patients with HF are presented in Figure 1. In a total of 108 included patients, the mean (SD) age was 70 (12) years, 61% were men, 59% had hypertension, and 33% had diabetes. Dyslipidemia, arrhythmia, and renal disease were less common (<10%). In addition, 22% had at least one hospitalization for HF within the previous 12 months, and two-thirds (66.0%) had at least one sign or symptom of congestion, such as dyspnea, leg edema, and general fatigue.

Nearly half of the patients (40%) had not consulted their cardiologist within the last 6 months. More than half of the patients were unsure whether they had HF (56.5%) and lacked knowledge regarding the self-assessment of symptoms of congestion (57.4%). One-quarter (25%) were uncertain as to the purpose of HF medications.

Lifestyle changes recommended in HF (regular physical activity, adapted daily eating habits) were followed by approximately 60% of patients.

The large majority of patients (89%) did not undergo an HF-related education program.

## 4. Discussion

In the present study, data stemming from unselected patients in pharmacies show that a large proportion of HF patients lacked HF-specific knowledge despite the presence of significant degrees of congestion/symptoms and that a sizeable proportion of these patients had poor access to specialized HF care.

Successful self-management is a key factor in improving the quality of care in patients with HF [6]. Nonetheless, we observed that more than half of unselected patients from general communities lacked basic knowledge on how to self-manage HF. The most worrisome finding was possibly the low level of knowledge regarding congestion despite the presence of significant signs and/or symptoms of congestion in two-thirds of patients with HF. These findings suggest a substantial gap between recommended patient knowledge in the field of HF and what is actually achieved in the “real world”.

In previously published data, intervention in pharmacies to optimize medical management was found to improve patient outcomes in HF with reduced ejection fraction [7,8]. This success may suggest that repeated brief periods of patient education in pharmacies may have a positive effect. Importantly, our results suggest that empowering pharmacists might help reach patients with moderate access to dedicated education programs. This approach may help achieve optimal levels of patients’ education. The additional help of pharmacists appears useful given that limited resources are typically the main hurdle to HF management and/or education.

Taken together, these data highlight the major gap in patient knowledge related to HF and suggest that collaborative multidisciplinary approaches involving pharmacists could potentially improve HF management.

There were several limitations to our study. The relatively moderate sample size of the current study may limit the generalization of our findings. Further studies including a larger number of patients are warranted to better ascertain the levels of patient knowledge, awareness, adherence, and prognostic values in HF. Although we provided a training period for pharmacists, they were less likely to elicit signs and symptoms of congestion in HF compared with generally healthy workers. As this study was performed in a real-life setting of pharmacies, some patients may not be included because of logistical/time constraints. In addition, we lacked information regarding echocardiograms; however, this limitation is often seen in routine practice, and patients with HF generally require HF education regardless of degrees of cardiac dysfunction.

## 5. Conclusions

In patients with HF assessed in their pharmacies, a majority lacked fundamental knowledge regarding HF, such as self-assessment of congestion, possibly due to a minimal proportion of patients undergoing an HF education program. These results suggest that interventions led by pharmacies may help improve HF education coverage in patients who may have poor access to specialized care.

## Data Availability

Data is available upon reasonable request to the corresponding author.

## Supplementary Materials

The following are available online at https://www.mdpi.com/article/10.3390/jcm11030863/s1, Figure S1: Heart Failure Questionnaire.
