# Supplementary material for: Assessment of Patient Knowledge, Awareness, and Adherence in Heart Failure in a Real-Life Setting: Insights from Data Acquired in Pharmacies
Source: J Clin Med. 2022 Feb 7;11(3):863. doi: 10.3390/jcm11030863 (PMC8837130; doi:10.3390/jcm11030863)
Supplement: Supplementary file 1 [file jcm-11-00863-s001.zip › jcm-1527876-supplementary.pdf]

Supplementary Figure S1. Heart Failure Questionnaire

1/ Patient information

- Have you been diagnosed with heart failure?

- Medical history:

i) Hypertension; ii) Diabetes; iii) Others

- When was the last time you had a consult with a cardiologist?

i) <3 months; ii) 3 - 6 months ; iii) 6 - 12 months; iv) >1 years; v) Never

- Were you hospitalization for worsening heart failure within 1 year?

If answer yes, how many times?

2/ Heart Failure Knowledge

- Do you know the signs of worsening of your disease / signs of congestion?

Do you currently experience the following?

i) Dyspnea; ii) Increase in body weight (2-3 kg for several days); iii) Leg edema; iv) General fatigue

- Do the above symptoms limit your dairy physical activation?

i) Slight limitation; ii) Marked limitation; iii) Unable to carry on any physical activity, even at rest

- Do you regularly take exercise (walk, gardening, bicycle)?

- Do you adapt your food habits according to heart failure (i.e., water or salt intake)?

- Are you vaccinated? What vaccine have you received?

i) No; ii) Flu; iii) Community acquired pneumonia; iv) Both

- Do you generally consult a general doctor when you have acute symptoms (*i.e.,* runny nose, bronchitis, enteritis; tooth-related problem)?

3/ Patient education

- Have you ever participated in the following heart failure education program?

i) Therapeutic education program; ii) national post-discharge program; iii) Others
